# Efficacy of 2% Hydroxypropyl Methylcellulose and Bandage Contact Lens for the Management of Dry Eye Disease after Cataract Surgery

**DOI:** 10.1155/2024/8415425

**Published:** 2024-02-01

**Authors:** Tianyao Zhao, Xiaodan Jiang, Ran Hao, Yi Ding, Dalan Jing, Xuemin Li

**Affiliations:** ^1^Department of Ophthalmology, Peking University Third Hospital, Beijing, China; ^2^Beijing Tongren Eye Center, Beijing Tongren Hospital, Capital Medical University, Beijing, China; ^3^Medical Oncology College, Beijing Shijitan Hospital, Capital Medical University, Beijing, China

## Abstract

**Objectives:**

To investigate the effect of 2% hydroxypropyl methylcellulose (HPMC) and bandage contact lens (BCL) on dry eye disease after cataract surgery.

**Methods:**

This prospective randomized controlled trial included 63 eyes which were divided into the balanced salt solution (BSS), HPMC, BCL, and combined HPMC and BCL (H&B) groups. The Ocular Surface Disease Index (OSDI), tear meniscus height (TMH), and average tear break-up time were measured before cataract surgery and 30 days postoperatively. Differences in corneal nerve fiber (CNF) and dendritic cell (DC) density in various directions were evaluated and compared. The CNFs and DCs in central and infratemporal directions were observed using *in vivo* confocal microscopy. Data were evaluated using the Kruskal–Wallis rank-sum test and analysis of variance.

**Results:**

The differences in variations in OSDI and TMH after cataract surgery between the four groups were statistically significant (*P* < 0.05). The postoperative OSDI of the HPMC group decreased compared with their preoperative OSDI. A statistically significant difference in the variations of OSDI score was observed between the HPMC and other groups (*P* < 0.05). The postoperative variations in TMH in the HPMC group were significantly higher than those observed preoperatively and significantly differed between HPMC and BCL groups and between BCL and H&B groups (*P* < 0.05). Postoperatively, the density of corneal DCs decreased in BSS and HPMC groups and increased in BCL and H&B groups (*P* < 0.001).

**Conclusions:**

The application of 2% HPMC in cataract surgery has a certain effect on managing dry eye after cataract surgery. Although the use of BCLs after cataract surgery has some benefits, it may cause mild ocular surface inflammation. Nevertheless, using 2% HPMC with BCLs in the perioperative phase of cataract surgery can alleviate the subjective discomfort of patients and can safely and effectively replace eye patch after cataract surgery.

## 1. Introduction

As cataract surgery moves towards precision and minimally invasive techniques, patients' expectations for postoperative visual quality have increased. However, a growing number of studies have focused on dry eye symptoms such as dry eyes, tearing, and increased foreign body sensation in many patients for several months after surgery. Objective postoperative results of auxiliary examination related to dry eye also suggest a tendency for dry eye to worsen compared to the preoperative period, significantly affecting the patient's quality of life [1, 2]. Therefore, reducing dry eye and maintaining a healthy ocular surface after cataract surgery are critical issues that require attention in modern cataract surgery.

The main causes of dry eye after cataract surgery include intraoperative water washout of the corneal epithelium, bright light irritation from the microscope, damage to the eyelid margin from the lid opener, damage to the corneal nerve from the incision, and reduction of conjunctival cupped cells [3, 4]. The primary ingredient of corneal epithelial protectors is 2% hydroxypropyl methylcellulose (HPMC). Studies have shown that the application of HPMC during cataract surgery can not only ensure the clarity of the operative field and reduce the frequency of aqueous flushing of the cornea but also reduce the damage to the corneal epithelium from the surgery, thus reducing postoperative dry eye [5, 6]. Therapeutic bandage contact lenses (BCLs) have been widely used in various ocular conditions related to corneal epithelial injury repair. They have also been shown to relieve discomfort and promote wound healing after cataract surgery [7–10]. However, some studies have indicated that the use of BCLs may cause some degree of ocular surface inflammation [11]. Therefore, it remains to be proven whether BCLs are suitable for reducing dry eye after cataract surgery.

Systematic protocols for managing dry eye during the perioperative period of cataract surgery are still lacking. This study aimed to investigate whether the application of corneal protectants during cataract ultrasound emulsion aspiration and/or the postoperative use of bandage lenses instead of eye pads could reduce postoperative dry eye in patients. *In vivo* confocal microscopy was used to observe the morphological variations of the patient's cornea before and after surgery, providing a basis for developing a reasonable program.

## 2. Methods

### 2.1. Patients

**Figure 1:**
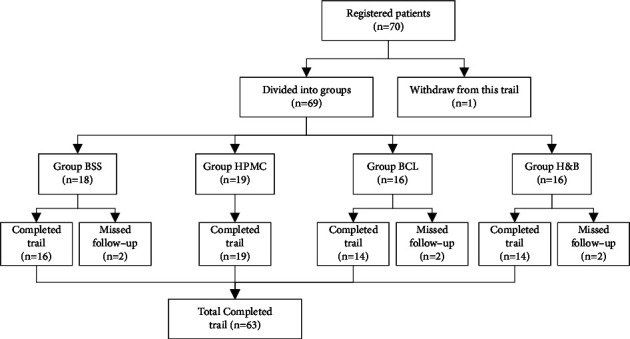
Flowchart describing the progress of patients through the trial. BSS: balanced salt solution; HPMC: hydroxypropyl methylcellulose; BCL: bandage contact lens; H&B: combined hydroxypropyl methylcellulose and bandage contact lens.

A total of 70 patients were recruited for the study. Before the start of the study, one patient withdrew from the study for personal reasons. It is inevitable that 6 patients were lost during the 30-day follow-up after surgery (Figure 1). Therefore, this study finally included 63 patients with age-related cataracts (63 eyes; average age, 67.68 ± 6.1 years; age range, 55–85 years) who underwent phacoemulsification combined with intraocular lens implantation in the Third Hospital of Peking University from March to July 2021. Patients with other eye conditions, including eye trauma, prior eye surgery, entropion and trichiasis, lacrimal passage illnesses, keratopathy, glaucoma, and uveitis, and those who used any topical eye drops before surgery, were excluded. Patients were excluded if they had a history of wearing contact lenses within one year. Patients with severe diabetes, thyroid disorders, and other systemic conditions such as autoimmune diseases that might lead to dry eye, as well as those with difficulties during or after the operation, were also excluded.

This study followed the tenets of the Declaration of Helsinki. The study's nature, objectives, content, and possible consequences were explained to the participants, and they provided written informed consent. The Peking University Third Hospital's Ethics Committee approved this investigation.

### 2.2. Surgical Procedure

All cataract ultrasonic emulsion aspiration combined with IOL implantation operations were performed by the same experienced ophthalmologist by using the same phacoemulsification instrument (Infiniti; Alcon Laboratories, Inc, Fort Worth, TX, USA). A 3.2-mm transparent corneal tunnel incision at 11'O clock was used for all patients. Surface anesthesia was performed with 2 doses of 0.4% oxybuprocaine hydrochloride eye drops (Santen Pharmaceutical Co., Ltd.), 1 drop each, preoperatively. There was a 3-minute interval between the two doses. The surgical eyes and surrounding accessories were disinfected with 0.5% povidone-iodine. The patients were divided into four groups according to whether they used 2% HPMC and BCL in the perioperative period of cataract surgery. For the balanced salt solution (BSS) group, the cornea was moistened with BSS when the cornea was dry during the operation, and an eye patch was used to cover the eyes after the operation. For the HPMC group, the entire cornea was equally coated with 2% HPMC before surgery, and the eye patch was used to cover the eyes after the operation. For the BCL group, the cornea was wetted with BSS when it became dry during the operation, and they were fitted with bandage contact lenses (Senofilcon A, Acuvue Oasys Hydraclear Plus (®); Johnson & Johnson, Jacksonville, FL) after the operation. They were removed at 24 h postoperatively. For the combined HPMC and BCL (H&B) group, the entire cornea was completely coated with 2% HPMC before surgery, and silicone hydrogel contact lenses were used after the operation. They were removed at 24 h postoperatively.

In the HPMC and H&B groups, a clear visual field and a moist cornea were always maintained during the procedure. Except for the need to rinse off the remaining HPMC with a small amount of BSS solution at the end of the surgery, no corneal wetting was required. The operation's overall duration was timed from the moment the eyelid opener was used until the moment it was removed at the end of the procedure. The operation's duration, cumulative dissipated energy, and BSS consumption data used for each group of moist corneas were recorded. Following surgery, eye drops containing 0.5% levofloxacin and 1% prednisolone acetate were administered 4 times per day in the first week, 3 times per day in the second week, 2 times per day in the third week, and once per day in the fourth week.

### 2.3. Clinical Evaluation

The best corrected visual acuity examination, intraocular pressure examination, slit-lamp biomicroscopy, corneal fluorescein staining (CFS), ocular B-ultrasound examination, and IOLMaster (Zeiss, Oberkochen, Germany) examination were performed preoperatively. In addition, patients underwent *in vivo* confocal microscopy evaluation 3 days before and 30 days after surgery. Each examination was performed by the same physician according to a standardized procedure.

### 2.4. Dry Eye Assessment

The Ocular Surface Disease Index (OSDI) of the patients was assessed, and they underwent CFS before surgery and 30 days after surgery [12]. Patients were asked a total of 12 questions in detail about their symptoms, impact on their lives, and environmental triggers in the last 1 week. The score for each question ranges from 0 to 4, depending on the frequency. The final score was the sum of the scores of all questions divided by the total number of questions multiplied by 25. A higher score indicated a higher degree of symptoms.

Keratograph 5M (OCULUS Optikgeräte GmbH, Wetzlar, Germany) was used to assess the average tear film break-up time (ATBUT) and tear meniscus height (TMH). The participants were asked to view the red light in the Placido ring, blink twice, and then immediately open their eyes and examined quickly. Their jaw was placed on the instrument's jaw support, and the height was adjusted so that the eyes were aligned with the Placido ring. To examine the meibomian gland, the eyelid had to be turned over to reveal the conjunctival surface of the eyelid, and the meibomian gland was photographed when it was clearly visible. The patients were asked to close their eyes and relax once the procedure was over. The evaluation was repeated every 10 minutes, and the average of 3 evaluations was obtained for each eye.

### 2.5. *In Vivo* Confocal Microscopy

Using the manual mode of an *in vivo* confocal microscope (Heidelberg Retina Tomography III Rostock Cornea Module; Heidelberg Engineering, Heidelberg, Germany), the center and edge of the cornea were photographed. Only images in the 400 *μ*m × 400 *μ*m (384 × 384 pixels) range were obtained. The test's safety warnings were discussed with the participants. One drop of 0.4% oxybuprocaine hydrochloride eye drops was applied to each eye, and the patients' side eyes were evaluated. During the inspection, each layer of the cornea's center and periphery was photographed separately, with the focal length ranging from shallow to deep, and the observation ranged from the corneal epithelium to the corneal endothelium. One drop of ofloxacin eye drops was applied after the examination to avoid infection. The density and morphology of the corneal nerves and cells were examined by using ImageJ V1.8.0.112 (National Institutes of Health, Bethesda, MD, USA) after 3 high-quality, distinct, and non-overlapping images of the corneal nerve vortex and periphery (below the temporal region) were chosen [11, 13–15]. Each parameter was examined by 2 workers, who then calculated the average.

### 2.6. Statistical Analysis

The data were analyzed using SPSS version 27.0 (IBM Corp., Armonk, NY, USA). Normally distributed data were expressed as mean ± standard deviation, whereas nonnormally distributed data were expressed as median and quartile, and grade-variable data were expressed as median (Q1 and Q3). The chi-square test was utilized to compare sex and eye type across groups. The least significant difference test was used to compare the two groups. Analysis of variance was used to compare groups with normally distributed data, whereas the Kruskal–Wallis test was used to compare groups with nonnormally distributed data. A p value of <0.05 was considered statistically significant.

## 3. Results

### 3.1. General Information

Table 1 presents the general information, duration of the procedure, cumulative dispersed energy, and preoperative outcomes for TMH, ATBUT, CFS, and OSDI. The findings demonstrated that a much smaller amount of BSS consumption for wetting cornea was used in the HPMC group than in the other groups. Other parameters did not significantly differ among the 4 groups (*P* > 0.05). Furthermore, no postoperative complications were observed during the 1-month follow-up.

### 3.2. Comparison of Dry Eye-Related Results

#### 3.2.1. Ocular Surface Disease Index

**Figure 2:**
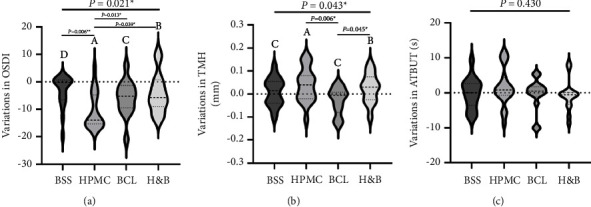
Comparison of the variations in dry eye-related parameters between the four groups (BSS, HPMC, BCL, and H&B) before and after cataract surgery: (a) variations in the OSDI, (b) variations in the TMH, and (c) variations in the ATBUT. BSS: balanced salt solution; HPMC: hydroxypropyl methylcellulose; BCL: bandage contact lens; H&B: combined HPMC and BCL; OSDI: Ocular Surface Disease Index; TMH: tear meniscus height; ATBUT: average tear film break-up time.

The results showed that the difference in the variations of postoperative OSDI scores among the four groups of patients was statistically significant (*P*=0.021) (Figure 2(a)). In addition, multiple comparisons showed statistically significant differences in the variations of postoperative OSDI scores between the HPMC group and all other groups (*P* < 0.05).

#### 3.2.2. Tear Meniscus Height

The difference in the variation in postoperative TMH between the four groups was statistically significant (*P* = 0.043). After pairwise comparisons, there was a statistically significant difference in the variations of TMH between the HPMC group and the BCL group, as well as between the BCL group and the H&B group (*P* < 0.05). However, there was no statistically significant difference in the variations of TMH between other groups (*P* > 0.05) (Figure2(b)).

#### 3.2.3. Average Tear Film Break-Up Time

There was no discernible difference in the variation of ATBUT among the 4 groups (*P*=0.430) (Figure 2(c)).

### 3.3. Comparison of Results of *In Vivo* Confocal Microscope-Related Parameters

#### 3.3.1. Corneal Epithelial and Basal Cells

The density of the central and peripheral corneal epithelial and basal cells before and after cataract surgery of the groups is presented in Table 2. There was no appreciable variation in the density of central and peripheral corneal epithelial and basal cells among the groups preoperatively or postoperatively (*P* > 0.05). The density of the corneal basal and epithelial cells did not differ among the 4 groups preoperatively or postoperatively (*P* > 0.05).

#### 3.3.2. Corneal Dendritic Cells

Table 3 presents the density of peripheral dendritic cells (DCs) and corneal vortex of the 4 groups preoperatively and postoperatively. The density of corneal DCs decreased in the BSS and HPMC groups, whereas it increased in the BCL and H&B groups, and the difference was statistically significant (*P* < 0.001). The pairwise comparison revealed significant differences in corneal DC density between the BSS and HPMC groups (*P* > 0.05), but there was no difference between the BSS and HPMC groups (*P* < 0.05).

The density of mature DCs in the corneal whirlpool area decreased in the BSS group, remained unchanged in the HPMC group, and decreased in the BCL and H&B groups. There was a significant difference in the density of mature DCs among the 4 groups (*P*=0.002). There was a significant difference in the density of mature DCs in the corneal peripheral area among the 4 groups (*P* < 0.001), with the BSS and HPMC groups having lower postoperative values than preoperative values, and there was no significant difference between the 2 groups (*P* > 0.05). The BCL and H&B groups had significantly higher postoperative values than preoperative values, especially the BCL group, and the difference between the 2 groups was statistically significant (*P*=0.011).

#### 3.3.3. Corneal Endothelial Cells

Before cataract surgery, there was no discernible difference among the 4 groups in terms of density of central and peripheral corneal endothelial cells (*P* > 0.05). However, after cataract surgery, there were notable variations in endothelial cell densities in the central cornea among the 4 groups (*P*=0.016). The density of corneal endothelial cells in the H&B group was higher than that in the HPMC and BCL groups (*P*=0.004 and *P*=0.07, respectively). Nevertheless, there were no differences in the variations in corneal endothelial cell density between the four groups in different areas before and after surgery (*P* > 0.05).

### 3.4. Analysis of the Corneal Nerve

#### 3.4.1. Corneal Nerve Fiber Density

Table 4 presents the corneal nerve fiber density (CNFD) of the 4 groups preoperatively and postoperatively. The CNFD in the corneal vortex area of the 4 groups decreased postoperatively, and the difference was statistically significant (*P*=0.007). The variations in CNFD in the corneal vortex area in the HPMC and H&B groups were smaller than those in the BSS and BCL groups (*P* < 0.05).

#### 3.4.2. Corneal Nerve Total Branch Density

As shown in Table 4, the corneal nerve total branch density (CTBD) in the corneal vortex area decreased in the 4 groups postoperatively, but the difference was not significant among groups (*P* > 0.05). However, the change trend of the average value was the same as that of CNFD in the corneal vortex area. There were significant differences in CTBD in the corneal peripheral area among the 4 groups postoperatively (*P* < 0.001).

The total CTBDs in the peripheral corneal area of the HPMC and H&B groups were higher than those preoperatively. In contrast, the total CTBDs in the peripheral corneal area of the BCL and H&B groups were significantly lower than those preoperatively, and the difference was statistically significant compared with the BSS and HPMC groups (*P* < 0.05).

## 4. Discussion

Although surgeons are increasingly focusing on issues with the ocular surface after cataract surgery, most surgeons can only treat postoperative dry eye symptoms with artificial tears. The currently available corneal wetting agents on the market have HPMC as the primary ingredient to keep the cornea moist during surgery and to prevent recurrent scouring of the corneal epithelium by water flow while improving optical clarity [6]. This is undoubtedly safer for cataract surgery. The results of the research by Yu et al. [5] showed that 2% HPMC has a certain effect on managing dry eye after cataract surgery. Nevertheless, a study has demonstrated that proper BCL administration after cataract surgery also contributes to the successful management of dry eye following cataract surgery [12]. Some studies have also shown that the application of BCL after pterygium excision or Müller's muscle-conjunctival resection seems effective in reducing postoperative pain and eye-stinging [7–9]. Our study confirms that HPMC and BCLs can be safely used in the perioperative phase of cataract surgery. We also confirmed that HPMC can effectively relieve the signs and symptoms of dry eye after cataract surgery, has a protective effect on corneal nerves, and is effective in reducing corneal inflammation caused by BCLs.

First, we observed that the OSDI scores of the HPMC, BCL, and H&B groups decreased by different degrees 1 month postoperatively compared with preoperative values. The postoperative OSDI score of the HPMC group was much lower than the preoperative score, which implies that the administration of 2% HPMC alone or in conjunction with BCLs can relieve subjective symptoms after cataract surgery, and the outcomes were consistent with those of other trials. Patients who use 2% HPMC during cataract surgery in conjunction with eye patch after cataract surgery seem to have better postoperative comfort than that offered by other methods [5, 12]. In our study, there was no significant difference in the ATBUT between the four groups preoperatively and postoperatively. It may be because the stability of tear film depends on the lipid and mucus layers to a greater extent, whereas corneal protectants and BCLs can only protect the corneal region but not the health of the meibomian gland or the number of conjunctival goblet cells [16, 17]. That is to say, only using corneal protectants during operation or BCLs after operation to protect the cornea may not improve the quality of tears to achieve the purpose of relieving dry eye postoperatively.

In addition, *in vivo* confocal microscopy was utilized to track variations in indices related to the cornea 30 days following cataract surgery. This allowed researchers to further examine the potential mechanisms by which different interventions may affect dry eye after cataract surgery. The first main branch of the trigeminal nerve, which is primarily responsible for preserving ocular surface sensation and epithelial integrity, has many nerve terminals in the cornea [18, 19]. In this study, we discovered that in each group, 30 days after cataract surgery, the CNFD and CTBD of the corneal nerve vortex decreased. These findings are in line with those of the literature on variations in the corneal nerve following cataract surgery [20, 21]. In addition, the reduction in CNFD and CTBD 1 month after operation in the 2 groups of patients who used 2% HPMC during operation in this study was significantly smaller than that in the other two groups, indicating that CNFD was highly correlated with corneal sensitivity [19]. In other words, the use of 2% HPMC during cataract surgery may lessen damage to CNFs while maintaining a certain level of corneal sensitivity and tear secretion. As a result, patients have more postoperative comfort.

DCs are efficient antigen-presenting cells that are crucial for controlling inflammation and immunity [6]. In our study, we found that the density and activation degree of DCs in the two groups of patients using BCLs increased 1 month postoperatively compared with that preoperatively. Alzahrani et al. [11] observed that the increase and activation of corneal DCs and the decrease of CNFD in patients with mild to moderate dry eye after wearing soft contact lenses may lead to increased discomfort and aggravation of dry eye. This is consistent with the results of our study. However, a study has shown that wearing BCLs has no effect on the corneal nerves or DCs [22]. We speculate that this may be related to the fact that the participants included in this study are elder patients with mild dry eye, whereas the previous participants were healthy young people. A study has shown that the corneal nerves and DCs can recover to their original levels after discontinuing the wearing of BCLs for 3 months [11]. Therefore, the inflammatory reaction caused by short-term use of BCLs is reversible. In addition, one of the two patient groups that used BCLs following surgery showed lower DC density and activation 1 month later than the group that did not use BCLs. Before the procedure, there was no distinction between the two groups, suggesting that the use of corneal protective agents can reduce the ocular surface inflammatory reaction caused by BCLs.

Despite the possibility of some ocular surface inflammation, the use of BCLs following cataract surgery provides benefits. First, patients with low vision can avoid the hassle of concealing their healthy eyes following surgery by using BCLs instead of eye patches [7, 8]. The use of BCLs ensures that the majority of the patients' usual activities following surgery and their ability to perform binocular vision tasks are unaffected. In addition, the use of BCLs can also avoid some unexpected situations. For example, there have been cases of patients with central retinal artery occlusion occurring within 1 day after cataract surgery or other intraocular surgery [23, 24]. Although the probability of such events is low, central retinal artery occlusion is a vascular disease caused by temporary occlusion of the retinal artery that can lead to retinal infarction and significant visual loss if not treated in time [25]. Wearing BCL after cataract surgery can help patients feel changes in their vision more promptly and detect any abnormalities in their eyes in a timely manner. This could lower the frequency of severe adverse effects following cataract surgery. Second, the use of BCLs may reduce the risk of endophthalmitis. There are bacteria in the meibomian gland of both healthy individuals and those with meibomian gland dysfunction, and the composition of these bacteria is more complex in patients with meibomian gland dysfunction, according to studies [26, 27]. The incision after cataract surgery is frequently made above the cornea. BCLs usage can successfully prevent contact and friction between the meibomian gland and the incision, thus protecting the corneal epithelium [12]. Therefore, it can reduce the probability of endophthalmitis occurring. Our prior research provided more evidence that BCLs can be administered to patients with cataracts after cataract surgery in a safe and efficient manner [13].

This study has some limitations. First, the number of study participants included in the sample was small; thus, the sample size needs to be further increased in future studies. Second, there was a sex imbalance in the study population, which might be explained by the fact that more female patients underwent cataract surgery than male patients throughout the study period. Further research must address these difficulties.

## 5. Conclusion

In conclusion, 2% HPMC is safe to use in cataract surgery. The use of 2% HPMC can alleviate or even improve the signs and symptoms of dry eye after cataract surgery and can effectively alleviate the surgical injury to the corneal nerve. In addition, its use can also effectively reduce the corneal inflammatory reaction caused by wearing BCLs. Therefore, the use of 2% HPMC is recommended for patients who must use BCLs after cataract surgery.

## Figures and Tables

**Table 1:** Comparison of basic information, preoperative parameters, and operation conditions.

Parameters	BSS	HPMC	BCL	H&B	*P* value
Eyes	16	19	14	14	
Age (years)	69.44 ± 4.97	67.74 ± 6.07	67.79 ± 7.98	65.50 ± 5.05	0.381
Sex (M/F)	2 : 14	4 : 15	3 : 11	2 : 12	0.875
Eye (OD/OS)	9 : 7	7 : 12	6 : 8	7 : 7	0.691
Operation time (s)	325.06 ± 56.59	330.42 ± 52.95	331.79 ± 65.41	302.79 ± 47.75	0.474
CDE	3.02 ± 1.03	3.07 ± 1.41	3.06 ± 1.02	3.08 ± 1.45	0.999
BSS consumption (mL)	4.00 ± 0.63	1.05 ± 0.23	3.93 ± 0.73	3.71 ± 0.83	0.002
TMH (mm)	0.18 ± 0.03	0.17 ± 0.06	0.20 ± 0.07	0.17 ± 0.06	0.259
BUT (s)	9.57 ± 5.98	9.13 ± 5.67	8.40 ± 5.97	9.33 ± 7.12	0.864
CFS	0.00 ± 0.00	0.00 ± 0.00	0.00 ± 0.00	0.00 ± 0.00	0.857
OSDI	22.41 ± 10.99	23.80 ± 9.24	21.32 ± 12.10	21.54 ± 12.68	0.620

BSS, balanced salt solution; HPMC, hydroxypropyl methylcellulose; BCL, bandage contact lens; H&B, combined hydroxypropyl methylcellulose and bandage contact lens; OD, right eye; OS, left eye; CDE, cumulative dissipated energy; TMH, tear meniscus height; BUT, break-up time; CFS, corneal fluorescein staining; OSDI, Ocular Surface Disease Index; M, male; F, female.

**Table 2:** Cell density of the corneal tissues.

Groups	Eyes	Cell density of the corneal tissues (cells/mm^2^)					
		Central cornea			Peripheral cornea		
		Preoperative	Postoperative	Variation	Preoperative	Postoperative	Variation
*Corneal epithelial cells*							
BSS	16	4953.00 ± 583.53	4875.00 ± 539.51	−78.00 ± 884.66	4990.94 ± 588.24	5047.25 ± 651.93	56.31 ± 956.68
HPMC	19	4561.14 ± 817.64	4642.86 ± 715.33	81.71 ± 927.71	4710.59 ± 775.01	4825.60 ± 677.77	52.00 ± 641.09
BCL	14	4813.71 ± 498.64	5044.00 ± 524.32	230.29 ± 654.91	4820.42 ± 532.86	5148.00 ± 470.36	327.58 ± 654.60
H&B	14	4862.00 ± 618.64	5083.00 ± 335.56	221.00 ± 670.41	4984.57 ± 659.60	5292.00 ± 584.53	396.00 ± 802.09
*F*		0.507	1.003	0.259	0.683	1.450	0.733
*P* value		0.681	0.407	0.855	0.566	0.239	0.537
*Corneal basal cells*							
BSS	16	5843.50 ± 709.15	5921.50 ± 978.55	78.00 ± 871.46	6006.00 ± 628.03	6116.50 ± 818.65	110.50 ± 775.68
HPMC	19	5057.46 ± 1368.59	5526.86 ± 541.83	469.39 ± 997.00	5382.51 ± 936.38	5622.50 ± 873.20	286.61 ± 851.80
BCL	14	5720.00 ± 462.19	6076.57 ± 418.62	356.57 ± 545.73	5785.96 ± 663.38	6153.33 ± 882.01	367.38 ± 566.20
H&B	14	5791.50 ± 612.09	6480.50 ± 858.03	314.00 ± 882.14	5872.29 ± 616.78	6328.62 ± 784.47	516.62 ± 983.52
*F*		1.356	2.067	0.290	1.799	1.943	0.476
*P* value		0.278	0.129	0.832	0.158	0.134	0.700
*Corneal endothelial cells*							
BSS	16	2529.63 ± 157.94	2483.88 ± 330.20	−45.75 ± 420.54	2510.44 ± 313.91	2362.81 ± 300.54	−147.62 ± 437.81
HPMC	19	2390.86 ± 499.46	2249.57 ± 304.99	−141.29 ± 402.19	2479.95 ± 377.81	2217.16 ± 379.25	−262.79 ± 360.53
BCL	14	2557.86 ± 315.39	2283.71 ± 198.58	−274.14 ± 401.21	2565.79 ± 346.66	2315.07 ± 373.21	−250.71 ± 534.73
H&B	14	2806.38 ± 298.79	2769.88 ± 406.20	−36.50 ± 506.12	2702.86 ± 361.19	2383.43 ± 619.37	−319.43 ± 593.36
*F*		2.049	4.154	0.470	1.202	0.519	0.343
*P* value		0.132	0.016^*∗*^	0.706	0.317	0.671	0.794

BSS, balanced salt solution; HPMC, hydroxypropyl methylcellulose; BCL, bandage contact lens; H&B, combined hydroxypropyl methylcellulose and bandage contact lens. ^∗^*P* < 0.05.

**Table 3:** Comparison of dendritic cell-related parameters.

		Central cornea			Peripheral cornea		
		Preoperative	Postoperative	Variation	Preoperative	Postoperative	Variation
*Total cells (cells/mm* ^*2*^)							
BSS	15	27.54 ± 8.09	11.00 ± 6.27	−16.54 ± 7.30	13.67 ± 5.69	3.91 ± 5.04	−9.77 ± 6.02
HPMC	17	23.03 ± 8.60	19.08 ± 15.66	−3.95 ± 12.01	16.78 ± 4.68	4.93 ± 6.45	−11.84 ± 2.89
BCL	14	22.77 ± 7.19	39.74 ± 14.32	16.97 ± 8.65	12.05 ± 9.32	20.44 ± 15.81	8.39 ± 6.49
H&B	14	25.67 ± 9.10	32.59 ± 25.26	6.92 ± 27.55	12.05 ± 10.24	13.39 ± 13.62	1.34 ± 17.19
*F*/*H*		1.199	9.413	12.954	1.980	8.022	16.438
*P* value		0.318	<0.001^*∗∗*^	<0.001^*∗∗*^	0.577	0.011^*∗*^	<0.001^*∗∗*^
*DCs with dendrites (cells/mm* ^*2*^)							
BSS	15	7.03 ± 4.49	4.55 ± 5.21	−2.48 ± 7.97	5.86 ± 4.25	1.56 ± 2.80	−4.30 ± 6.74
HPMC	17	5.59 ± 8.04	6.25 ± 9.32	0.66 ± 1.97	5.26 ± 6.34	0.99 ± 2.34	−4.28 ± 4.68
BCL	14	5.36 ± 5.40	12.59 ± 6.99	7.23 ± 7.01	4.91 ± 4.37	10.85 ± 8.39	5.94 ± 5.08
H&B	14	4.46 ± 4.54	13.39 ± 26.61	8.93 ± 6.03	4.02 ± 5.27	4.02 ± 5.26	0.00 ± 7.35
*F*/*H*		0.479	8.021	11.486	0.325	12.192	9.916
*P* value		0.698	0.011^*∗*^	0.002^*∗*^	0.807	<0.001^*∗∗*^	<0.001^*∗∗*^

BSS, balanced salt solution; HPMC, hydroxypropyl methylcellulose; BCL, bandage contact lens; H&B, combined hydroxypropyl methylcellulose and bandage contact lens; DCs, dendritic cells. ^∗^*P* < 0.05; ^∗∗^*P* < 0.001.

**Table 4:** Comparison of corneal nerve fibers.

		Central cornea			Peripheral cornea		
		Preoperative	Postoperative	Variation	Preoperative	Postoperative	Variation
*CNFD (n/mm^2^)*							
BSS	15	18.51 ± 2.15	14.16 ± 2.22	−3.35 ± 2.67	13.07 ± 3.29	10.73 ± 2.77	−2.35 ± 4.26
HPMC	17	17.36 ± 3.42	17.06 ± 2.41	−0.30 ± 3.66	13.51 ± 2.42	13.71 ± 3.46	0.19 ± 3.23
BCL	14	19.07 ± 3.61	15.81 ± 3.09	−3.26 ± 2.09	13.54 ± 3.96	11.32 ± 5.62	−2.22 ± 2.68
H&B	14	17.50 ± 2.94	16.81 ± 3.75	−0.69 ± 3.21	12.87 ± 4.23	11.47 ± 0.96	−1.40 ± 4.28
*H*/*F*		1.063	1.468	4.522	0.133	2.246	1.705
*P* value		0.372	0.233	0.007^*∗*^	0.940	0.336	0.176
*CTBD (n/mm^2^)*							
BSS	16	47.74 ± 9.42	42.87 ± 11.49	−4.77 ± 17.65	20.40 ± 18.72	14.49 ± 13.11	−5.91 ± 12.99
HPMC	19	49.19 ± 12.07	47.74 ± 16.10	−1.45 ± 14.22	18.72 ± 9.77	25.32 ± 5.86	8.68 ± 13.40
BCL	14	51.62 ± 18.50	31.16 ± 17.54	−20.46 ± 27.13	25.11 ± 18.03	15.34 ± 6.05	−9.77 ± 15.48
H&B	14	53.94 ± 5.37	53.94 ± 41.63	0.01 ± 43.64	21.39 ± 17.21	31.62 ± 28.53	10.23 ± 15.26
*F*/*H*		0.730	2.303	1.724	0.410	4.00	7.547
*P* value		0.203	0.089	0.218	0.746	0.025^*∗*^	<0.001^*∗∗*^

BSS, balanced salt solution; HPMC, hydroxypropyl methylcellulose; BCL, bandage contact lens; H&B, combined hydroxypropyl methylcellulose and bandage contact lens; CNFD, corneal nerve fiber density; CTBD, corneal nerve total branch density. ^∗^*P* < 0.05; ^∗∗^*P* < 0.001.

## Data Availability

The datasets used and/or analysed during the current study available from the corresponding author on reasonable request.

## Abbreviations

HPMC: Hydroxypropyl methylcellulose
BCLs: Bandage contact lenses
H&B: Combined HPMC and BCL
OSDI: Ocular Surface Disease Index
CFS: Corneal fluorescein staining
CDE: Cumulative dissipated energy
BSS: Balanced salt solution
ATBUT: Average tear film break-up time
TMH: Tear meniscal height
DCs: Dendritic cells
CNFD: Corneal nerve fiber density
CTBD: Corneal nerve total branch density.

## References

[1] Sajnani R., Raia S., Gibbons A. (2018). Epidemiology of persistent postsurgical pain manifesting as dry eye-like symptoms after cataract surgery. *Cornea*.

[2] Choi Y., Park S., Jun I. (2018). Perioperative ocular parameters associated with persistent dry eye symptoms after cataract surgery. *Cornea*.

[3] Miura M., Inomata T., Nakamura M. (2022). Prevalence and characteristics of dry eye disease after cataract surgery: a systematic review and meta-analysis. *Ophthalmology and therapy*.

[4] Lu Q., Lu Y., Zhu X. (2021). Dry eye and phacoemulsification cataract surgery: a systematic review and meta-analysis. *Frontiers of Medicine*.

[5] Yu X., Wang D., Chang P. (2017). The effect of three intraoperative corneal wetting solutions on dry eye after cataract surgery. *Chin J Optom Ophthalmol Vis Sci*.

[6] Chen Y., Hirnschall N., Findl O. (2011). Comparison of corneal wetting properties of viscous eye lubricant and balanced salt solution to maintain optical clarity during cataract surgery. *Journal of Cataract & Refractive Surgery*.

[7] Prat D., Zloto O., Ben Artsi E., Ben Simon G. (2018). Therapeutic contact lenses vs. tight bandage patching and pain following pterygium excision: a prospective randomized controlled study. *Graefe’s Archive for Clinical and Experimental Ophthalmology*.

[8] Daglioglu M., Coskun M., Ilhan N. (2014). The effects of soft contact lens use on cornea and patient’s recovery after autograft pterygium surgery. *Contact Lens and Anterior Eye: The Journal of the British Contact Lens Association*.

[9] Mangan M., Tekcan H., Yurttaser Ocak S. (2021). Efficacy of bandage contact lenses versus eye patching in early postoperative period of Müller’s muscle-conjunctival resection. *Ophthalmic Research*.

[10] Mangan M., Ocak S., Vural E., Yildiz E. (2021). Müller muscle-conjunctival resection with or without tarsectomy and combined with bandage contact lens use in ptosis patients with corneal graft. *Korean Journal of Ophthalmology*.

[11] Alzahrani Y., Colorado L., Pritchard N., Efron N. (2017). Longitudinal changes in Langerhans cell density of the cornea and conjunctiva in contact lens-induced dry eye. *Clinical and Experimental Optometry*.

[12] Schiffman R., Christianson M., Jacobsen G., Hirsch J., Reis B. (2000). Reliability and validity of the ocular surface disease index. *Archives of Ophthalmology*.

[13] Shetty R., Sethu S., Deshmukh R. (2016). Corneal dendritic cell density is associated with subbasal nerve plexus features, ocular surface disease index, and serum vitamin D in evaporative dry eye disease. *BioMed Research International*.

[14] Xu J., Chen P., Yu C., Liu Y., Hu S., Di G. (2021). In vivo confocal microscopic evaluation of corneal dendritic cell density and subbasal nerve parameters in dry eye patients: a systematic review and meta-analysis. *Frontiers of Medicine*.

[15] Han J., Liang H. H., Guo J. X., Guo J. (2021). Correlation of the retinopathy degree with the change of ocular surface and corneal nerve in patients with type 2 diabetes mellitus. *International Journal of Ophthalmology*.

[16] Li X., Hu L., Hu J., Wang W. (2007). Investigation of dry eye disease and analysis of the pathogenic factors in patients after cataract surgery. *Cornea*.

[17] Lum E., Golebiowski B., Swarbrick H. (2012). Mapping the corneal sub-basal nerve plexus in orthokeratology lens wear using in vivo laser scanning confocal microscopy. *Investigative Opthalmology & Visual Science*.

[18] Tepelus T., Chiu G., Huang J. (2017). Correlation between corneal innervation and inflammation evaluated with confocal microscopy and symptomatology in patients with dry eye syndromes: a preliminary study. *Graefe’s Archive for Clinical and Experimental Ophthalmology*.

[19] Giannaccare G., Bernabei F., Pellegrini M. (2021). Bilateral morphometric analysis of corneal sub-basal nerve plexus in patients undergoing unilateral cataract surgery: a preliminary in vivo confocal microscopy study. *British Journal of Ophthalmology*.

[20] Misra S., Goh Y., Patel D., Riley A., McGhee C. (2015). Corneal microstructural changes in nerve fiber, endothelial and epithelial density after cataract surgery in patients with diabetes mellitus. *Cornea*.

[21] D Z L H J C Y Y (2022). The effects of dry eye disease and contact lenses on corneal sub—epithelial nerve density with in vivo confocal microscopy observation. *Chinese Journal of Practical Ophthalmology*.

[22] Kocabeyoglu S., Colak D., Mocan M., Irkec M. (2019). Sensory adaptation to silicone hydrogel contact lens wear is not associated with alterations in the corneal subbasal nerve plexus. *Cornea*.

[23] Jung E., Park K., Woo S. (2015). Iatrogenic central retinal artery occlusion following retrobulbar anesthesia for intraocular surgery. *Korean Journal of Ophthalmology*.

[24] Swamy B., Merani R., Hunyor A. (2010). Central retinal artery occlusion after phacoemulsification. *Retinal Cases & Brief Reports*.

[25] Varma D., Cugati S., Lee A., Chen C. (2013). A review of central retinal artery occlusion: clinical presentation and management. *Eye*.

[26] Jiang X., Deng A., Yang J. (2018). Pathogens in the Meibomian gland and conjunctival sac: microbiome of normal subjects and patients with Meibomian gland dysfunction. *Infection and Drug Resistance*.

[27] Zhang S., He J., Niu T. (2017). Bacteriological profile of ocular surface flora in meibomian gland dysfunction. *Ocular Surface*.

